# Supplementary material for: From sequence to enzyme mechanism using multi-label machine learning
Source: BMC Bioinformatics. 2014 May 19;15:150. doi: 10.1186/1471-2105-15-150 (PMC4229970; doi:10.1186/1471-2105-15-150)
Supplement: Additional file 2 — Java code of ml2db. Additional file ml2db_code.tar.gz contains the Java source code to run the multi-label machine learning experiments and save the results to database. The code’s Javadoc is included. [file 1471-2105-15-150-S2.zip › additional file 2/ml2db/ml2db/doc/test/ml2db/dataharness/TestProjectParameters.html]

TestProjectParameters


JavaScript is disabled on your browser.


- Overview
- Package
- Class
- Use
- Tree
- Deprecated
- Index
- Help

- Prev Class
- Next Class

- Frames
- No Frames

- All Classes

- Summary:
- Nested |
- Field |
- Constr |
- Method

- Detail:
- Field |
- Constr |
- Method


test.ml2db.dataharness

## Class TestProjectParameters

- java.lang.Object
- - main.ml2db.ProjectParameters
  - - test.ml2db.dataharness.TestProjectParameters

- ---

    

  ```
  public class TestProjectParameters
  extends ProjectParameters
  ```

- - ### Field Summary

    Fields

    | Modifier and Type | Field and Description |
    | --- | --- |
    | `static java.lang.String` | `ARFF_DB_PROPS` |
    | `static java.lang.String` | `ARFF_FILES_PATH` |
    | `static java.lang.String` | `ARFF_PROPS_NO_XML` |
    | `static java.lang.String` | `ARFF_PROPS_NUMERIC_ATT` |
    | `static java.lang.String` | `ARFF_PROPS_PATH` |
    | `static java.lang.String` | `BASE_PATH` |
    | `static boolean` | `DEBUG` |
    | `static java.lang.String` | `FILTER_OLD_TEST_SET` |
    | `static java.lang.String` | `FILTER_TRAIN_SET` |
    | `static java.lang.String` | `INSTANCE_1_2` |
    | `static java.lang.String` | `INSTANCE_1_2_EMPTY` |
    | `static java.lang.String` | `INSTANCE_3` |
    | `static java.lang.String` | `ML_PROPS_PATH` |
    | `static java.lang.String` | `MODEL_FOLDER` |
    | `static java.lang.String` | `MODELS_PATH` |
    | `static java.lang.String` | `PLOTS_FOLDER` |
    | `static java.lang.String` | `PREDICTIONS_DATA` |
    | `static java.lang.String` | `PREDICTIONS_FOLDER` |
    | `static java.lang.String` | `PROPS_PATH` |
    | `static java.lang.String` | `RESULTS_DIRECTORY_ACTIVE_LEARNING` |
    | `static java.lang.String` | `RESULTS_FOLDER` |
    | `static java.lang.String` | `TEST_ARFF_PROPS_1` |
    | `static java.lang.String` | `TEST_ARFF_PROPS_2` |
    | `static java.lang.String` | `TEST_FIGURES_PATH` |
    | `static java.lang.String` | `TEST_MACHINE_LEARNING_PROPS` |
    | `static java.lang.String` | `TEST_REMOTE_MACHINE_LEARNING_PROPS` |

    - ### Fields inherited from class main.ml2db.ProjectParameters

      `ARFF_FILES_DIR, ARFF_TABLE, AUTHOR, DATA_LOCAL_DB, DATA_REMOTE_DB, EXPECTED_NUMBER_OF_UNIQUE_ATTRIBUTES, EXPECTED_NUMBER_OF_UNIQUE_INSTANCES, FIGURES_PATH, MACHINE_LEARNING_LOCAL_DB_PROPS, MACHINE_LEARNING_PROPS, MACHINE_LEARNING_REMOTE_DB_PROPS, MACIE_HOM_DB_PROPS, PREDICTIONS_PATH, PROJECT_NAME, RESULTS_DIRECTORY, SOFTWARE_PROJECT_NAME, STATISTICS_PATH, UNIPROT_DB_PROPS, VERSION, WEB_PAGE`
  - ### Constructor Summary

    Constructors

    Constructor and Description

    `TestProjectParameters()`
  - ### Method Summary

    Methods

    | Modifier and Type | Method and Description |
    | --- | --- |
    | `static DbManagers` | `getArffRealRestTestDbManagers()` |
    | `static DbManagers` | `getDbManagers()` |
    | `static uk.ac.standrews.utils.main.database.DbManager` | `getTestArffRecordsDbManager()` |
    | `static uk.ac.standrews.utils.main.database.DbManager` | `getTestDataDbManager()` |
    | `static uk.ac.standrews.utils.main.database.DbManager` | `getTestMachineLearningResultsDbManager()` |

    - ### Methods inherited from class main.ml2db.ProjectParameters

      `getLocalDbManagers, getRemoteDbManagers`
    - ### Methods inherited from class java.lang.Object

      `equals, getClass, hashCode, notify, notifyAll, toString, wait, wait, wait`

- - ### Field Detail


    - #### DEBUG

      ```
      public static final boolean DEBUG
      ```

      See Also:
      :   Constant Field Values


    - #### BASE\_PATH

      ```
      public static final java.lang.String BASE_PATH
      ```

      See Also:
      :   Constant Field Values


    - #### PREDICTIONS\_FOLDER

      ```
      public static final java.lang.String PREDICTIONS_FOLDER
      ```

      See Also:
      :   Constant Field Values


    - #### PREDICTIONS\_DATA

      ```
      public static final java.lang.String PREDICTIONS_DATA
      ```

      See Also:
      :   Constant Field Values


    - #### MODELS\_PATH

      ```
      public static final java.lang.String MODELS_PATH
      ```

      See Also:
      :   Constant Field Values


    - #### PLOTS\_FOLDER

      ```
      public static final java.lang.String PLOTS_FOLDER
      ```

      See Also:
      :   Constant Field Values


    - #### PROPS\_PATH

      ```
      public static final java.lang.String PROPS_PATH
      ```

      See Also:
      :   Constant Field Values


    - #### ARFF\_FILES\_PATH

      ```
      public static final java.lang.String ARFF_FILES_PATH
      ```

      See Also:
      :   Constant Field Values


    - #### ARFF\_PROPS\_PATH

      ```
      public static final java.lang.String ARFF_PROPS_PATH
      ```

      See Also:
      :   Constant Field Values


    - #### ML\_PROPS\_PATH

      ```
      public static final java.lang.String ML_PROPS_PATH
      ```

      See Also:
      :   Constant Field Values


    - #### ARFF\_PROPS\_NO\_XML

      ```
      public static final java.lang.String ARFF_PROPS_NO_XML
      ```

      See Also:
      :   Constant Field Values


    - #### TEST\_MACHINE\_LEARNING\_PROPS

      ```
      public static final java.lang.String TEST_MACHINE_LEARNING_PROPS
      ```

      See Also:
      :   Constant Field Values


    - #### TEST\_REMOTE\_MACHINE\_LEARNING\_PROPS

      ```
      public static java.lang.String TEST_REMOTE_MACHINE_LEARNING_PROPS
      ```


    - #### TEST\_ARFF\_PROPS\_1

      ```
      public static final java.lang.String TEST_ARFF_PROPS_1
      ```

      See Also:
      :   Constant Field Values


    - #### TEST\_ARFF\_PROPS\_2

      ```
      public static final java.lang.String TEST_ARFF_PROPS_2
      ```

      See Also:
      :   Constant Field Values


    - #### ARFF\_PROPS\_NUMERIC\_ATT

      ```
      public static final java.lang.String ARFF_PROPS_NUMERIC_ATT
      ```

      See Also:
      :   Constant Field Values


    - #### ARFF\_DB\_PROPS

      ```
      public static final java.lang.String ARFF_DB_PROPS
      ```

      See Also:
      :   Constant Field Values


    - #### RESULTS\_FOLDER

      ```
      public static final java.lang.String RESULTS_FOLDER
      ```

      See Also:
      :   Constant Field Values


    - #### RESULTS\_DIRECTORY\_ACTIVE\_LEARNING

      ```
      public static final java.lang.String RESULTS_DIRECTORY_ACTIVE_LEARNING
      ```

      See Also:
      :   Constant Field Values


    - #### MODEL\_FOLDER

      ```
      public static final java.lang.String MODEL_FOLDER
      ```

      See Also:
      :   Constant Field Values


    - #### FILTER\_OLD\_TEST\_SET

      ```
      public static final java.lang.String FILTER_OLD_TEST_SET
      ```

      See Also:
      :   Constant Field Values


    - #### FILTER\_TRAIN\_SET

      ```
      public static final java.lang.String FILTER_TRAIN_SET
      ```

      See Also:
      :   Constant Field Values


    - #### INSTANCE\_1\_2

      ```
      public static final java.lang.String INSTANCE_1_2
      ```

      See Also:
      :   Constant Field Values


    - #### INSTANCE\_1\_2\_EMPTY

      ```
      public static final java.lang.String INSTANCE_1_2_EMPTY
      ```

      See Also:
      :   Constant Field Values


    - #### INSTANCE\_3

      ```
      public static final java.lang.String INSTANCE_3
      ```

      See Also:
      :   Constant Field Values


    - #### TEST\_FIGURES\_PATH

      ```
      public static final java.lang.String TEST_FIGURES_PATH
      ```

      See Also:
      :   Constant Field Values
  - ### Constructor Detail


    - #### TestProjectParameters

      ```
      public TestProjectParameters()
      ```
  - ### Method Detail


    - #### getArffRealRestTestDbManagers

      ```
      public static DbManagers getArffRealRestTestDbManagers()
      ```

      Returns:


    - #### getDbManagers

      ```
      public static DbManagers getDbManagers()
      ```


    - #### getTestArffRecordsDbManager

      ```
      public static uk.ac.standrews.utils.main.database.DbManager getTestArffRecordsDbManager()
      ```


    - #### getTestDataDbManager

      ```
      public static uk.ac.standrews.utils.main.database.DbManager getTestDataDbManager()
      ```


    - #### getTestMachineLearningResultsDbManager

      ```
      public static uk.ac.standrews.utils.main.database.DbManager getTestMachineLearningResultsDbManager()
      ```


- Overview
- Package
- Class
- Use
- Tree
- Deprecated
- Index
- Help

- Prev Class
- Next Class

- Frames
- No Frames

- All Classes

- Summary:
- Nested |
- Field |
- Constr |
- Method

- Detail:
- Field |
- Constr |
- Method
